# Alternative Splicing in Self-Renewal of Embryonic Stem Cells

**DOI:** 10.4061/2011/560261

**Published:** 2011-06-09

**Authors:** Clara Y. Cheong, Thomas Lufkin

**Affiliations:** Stem Cell & Developmental Biology, Genome Institute of Singapore, 60 Biopolis Street, Singapore 138672

## Abstract

Much of embryonic stem cell biology has focused on transcriptional expression and regulation of genes that could mediate its unique potential in self-renewal or pluripotency. In alignment with our present understanding on the genetic, protein, and epigenetic factors that may direct cell fate, we present a short overview of the often overlooked contribution of alternative splice variants to regulatory diversity. Progressing beyond the limitations of a fixed genomic sequence, alternative splicing offers an additional layer of complexity to produce protein variants that may differ in function and localization that can direct embryonic stem cells to specific differentiation pathways. In light of the number of variants that can be produced at key ES cell genes alone, it is challenging to consider how much more multifaceted transcriptional regulation truly is, and if this can be captured more fully in future works.

## 1. Introduction

Embryonic stem cells (ESC or ES cells) are unique in their ability to self-renew and differentiate into specialized lineages representative of the three germ layers of an organism [1, 2]. First isolated from the inner cell mass (ICM) of a mouse blastocyst and subsequently in other species including human [3], ES cells have provided insight into the fundamental workings of otherwise inaccessible early developmental stages. Because of the pluripotent nature of ESCs, their potential to regenerate specific cell types is also of therapeutic interest. In this respect, strides have been made with the discovery of protein factors that were able to reprogram somatic cells into induced pluripotent stem cells (iPS cells) types that bear much similarity to ES cells [4, 5]. These reprogramming studies together with work in ES cells have provided a vast amount of insight into the pathways, mechanisms, and key transcription factors involved in pluripotency. In addition to transcriptional regulation, epigenetic mechanisms such as chromatin modifications are now known to aid in the activation/repression of developmental stage-specific genes [6–9]. Reasonably, much of our present understanding of ES cells has and will continue to arrive through comparative studies of pluripotent cells to its differentiated counterparts, though these comparisons require further refinement in light of the multiple mechanisms and subtle differences in protein complexes used in transcriptional regulation. Less widely understood are the complementary mechanisms that refine the transcriptional profile of ES cells, going beyond bulk gene expression levels to look at the transcript variants and alternative splicing (AS) that occurs at each gene.

More than 74% of human genes are known to undergo alternative splicing [10, 11], a phenomenon that can result in a combination of exons and/or untranslated 5′ and 3′ regions (UTRs) that differ from the canonical transcript. Alternatively spliced products can have implications on protein translation and RNA regulation [12], and aberrant splicing is responsible for up to 15% of human genetic disease caused by point mutations [13]. Correctly spliced, alternative transcripts can increase the diversity of the proteome through multiple splicing permutations, in a manner that does not require a concomitant increase in gene sequence. 

Mouse and human ES cells share many similarities including their dependence on key ES cell transcription factors Oct4, Sox2, and Nanog for pluripotency [14–17], existence of “bivalent” chromatin marks that repress differentiation-specific genes [18] and importance of Polycomb repressor complexes in transcriptional repression [19]. However, there are still discrepancies in the signaling pathways and requirements for specific transcription factors or micro-RNAs between mouse and human ES cells, the most well-documented being that of the LIF/Jak-STAT pathway signaling required for mouse but not human ES cells [20–22]. In somatic cell reprogramming, a related set of factors were found to be necessary for the generation of iPS cells. While OCT4/Oct4 and SOX2/Sox2 were deemed necessary for both mouse and human ES cells, additional mouse factors originally required were Klf4 and c-Myc, whereas the combination included NANOG and LIN28 in the case of human ES cells [4, 5]. Plausibly, the prevalence, yet underestimated impact of AS can serve to fine-tune our understanding of these species-specific differences, seen in the light of the increased functional dimensions presented by alternative transcripts.

In this paper, we highlight works related to alternative splicing of key ES cell transcription factors. Because of the paucity of AS variant data in ES cells, AS findings of these factors in other developmental stages may also give insights into the previously unexplored potential of these factors in ES cells. This paper also demonstrates the importance of examining splice variants despite their apparently subtle sequence differences.

## 2. Functional Implications of Alternative Splicing

Through expressed sequence tag (EST) databases for multiple tissues and species, a large percentage of mammalian genes (35–60%) were found to be alternatively spliced, by alignment of these EST sequences to the cDNA reference [23–25]. Though AS can occur anywhere across the transcribed sequence, the majority of splicing occurs within the coding region and can serve to increase the repertoire of proteins that may be utilized in the cell [24, 26, 27]. While an average of 2–5 AS transcripts are produced for each human gene involved in AS [12], an extreme example demonstrates the vast complexity allowed by AS; the *Drosophila *gene Dscam generates a potential 38000 isoforms, separated by developmental time and space [28]. 

Through the inclusion or exclusion of exons, protein domains are altered. This can result in changes to binding affinities, catalytic activity, stability, localization, or even posttranslational modifications [12]. 

Yet not all exon changes result in a new protein—some result in the degradation of the mRNA instead. Nonsense-mediated decay (NMD) of the mRNA occurs if a stop codon is encountered at a distance more than 50 base pairs from the 3′ most splice junction, due to the accumulation of NMD-associated Upf proteins at the 3′ end that are not removed by ribosomes as they traverse the mRNA sequence [29, 30]. While these unproductive splice products are unstable and not well understood, they are not uncommon. Approximately 30% of alternative exons can introduce frameshifts and stop codons into mRNA sequences, resulting in a substantial amount of what is perceived as inefficient transcription [26, 31, 32]. Surprisingly, a phylogenetic study of unproductive splice variants among primate species using the DNA polymerase POLB gene, suggests that these occur at varying frequencies among primates and are largely not conserved, yet the extent of AS variants found correlate closely with life expectancy, as well as age at first reproduction [33]. Through a mechanism as yet unknown, these noncoding protein splice forms may serve to regulate transcription, and its levels may also be tuned to reflect the amount of transcriptional control required [34, 35].

The means of AS resulting in alternative promoter usage at the 5′ and 3′ UTRs are also not well understood. However, a recent study of AS in mouse ES cells found that 12% of genes that they examined for alternative exon usage were mapped to alternative promoter sites [36]. Given the function of the 5′ region in transcriptional initiation, and the poly(A) tract in the 3′ end for mRNA stability, it is likely that the use of alternative UTRs can alter the half-time of an mRNA strand, and by extension, the amount of protein production from a functional sequence [37, 38].

## 3. Evolutionary Impact of Alternative Splicing

While genome-wide comparisons between human and mouse demonstrate high-sequence conservation (~90%) at canonical exons of orthologous genes [39, 40], only about 25% of alternative exons were conserved. However, when such AS exons were identified in an EST database of one species, the corresponding exon was likely to be found in the other species as well [40]. The authors attributed the inclusion of a particular exon in an EST database as indicative of a minimum expression level and found this congruent with a finding that ancient conserved regions correlated with more highly expressed genes [41]. It appears that the ~75% remainder of alternative nonconserved exons are expressed at lower levels and segregate in a tissue-specific manner in the species of origin, suggesting that these exons arose after divergence from a common ancestor. The large proportion of alternative exons with such features emphasizes the significance of AS in promoting genetic variation and possibly, speciation along with increasing organismal complexity. Extending this to the variation offered by AS transcripts between for tissue specificity, a separate study comparing mouse ES to hematopoietic stem cells found that ~30% of splice variants were unique to the mouse ES cell profile, although the functionality of these variants remains to be seen [42]. In line with the widely held view that strongly conserved sequences are indicative of core functions [43], ubiquitously expressed genes such as heterogeneous nuclear ribonucleoproteins (hnRNPs) show little AS and are located in ultraconserved regions (defined as 200 base pairs or greater of sequence with 100% identity between human, mouse, and rat) [44, 45]. In contrast, tissue-specific genes are likely to exhibit a larger number of AS variants with little cross-species homology [42] and face much less selection pressure against the insertion of Alu and other repeat elements that can lead to sequence “exonisation” that is permissive for species adaptation [46, 47].

## 4. Alternative Transcripts Can Direct ES Cell Differentiation

### 4.1. OCT4

In both mouse and human, the OCT4/Oct4 protein is a well-described transcription factor containing a POU domain that is able to bind a consensus octamer sequence on DNA [48–50]. Belonging to a group of closely related proteins of the Oct family, OCT4 is related to multiple pseudogenes, derived from earlier retrotransposition events into other chromosomes [51–54], as well as AS transcripts arising directly from the *Oct4/Pou5f1* locus [55, 56]. While OCT4 pseudogenes are expressed in human hematopoietic stem cells, it is uncertain if these pseudogenes are involved in ES cells [53, 54]. However, alternative splicing of *OCT4* is evident in ES cells, where *OCT4* is most highly expressed [15, 48]. The most commonly described transcript is *OCT4A*, which is translated into a full-length nuclear-localized OCT4 protein with an N- and C-terminal transactivation domain separated by a POU DNA-binding domain [48]. Interestingly, the primary shorter transcript, *OCT4B*, still contains the same downstream sequence as *OCT4A*, albeit with a shorter N-terminal domain that results from a skipped exon 1, but an extended 5′ end of exon 2 [55, 56]. Though a putative nuclear localization signal appears to be retained in the translated OCT4B protein, it is cytoplasmically located, in contrast to OCT4A [57].

Recently, a novel third transcript, *OCT4B1*, was identified as an ES cell-specific transcript of OCT4 and considered as a possible stemness marker, given its significant correlation with *NANOG* expression in ES and differentiated cell lines [56, 58]. While it is still uncertain if *OCT4B1* functions primarily as a transcript, or is translated into protein products, preliminary evidence from multiple groups suggests that *OCT4B1* can be spliced into the same products as *OCT4B*, all of which are cytoplasmically located [59, 60].

Furthering the diversity of output from a single locus, *OCT4* not only undergoes AS of mRNA transcripts, but goes a step further with different translations from a single mature mRNA form. Unusually, *OCT4B* and *OCT4B1* mRNA both contain 2 possible start codons, as well as an internal ribosomal entry site (IRES) formed in part by the extended 5′ end of exon 2 in OCT4B/B1 [55, 56, 61, 62]. This results in proteins of 265, 190, and 165 amino acids (a.a.) from *OCT4B/B1* although it cannot be excluded that the 164 a.a. long version is also translated from *OCT4A*, which also contains the downstream in-frame ATG codon in the 3′ end of exon 2. Structurally, the shorter OCT4 variants produced do not contain an N-terminal transactivation domain, but more importantly, lack the POU-S portion of the complete POU domain [50, 63]. Functionally, it appears that OCT4A and OCT4B variants may serve differently, since they localize to different cellular regions. OCT4A is found in the nucleus and is responsible for the well-known activities of OCT4 in ES cell self-renewal and pluripotency [64–66]. Conversely, the full OCT4B isoform (or OCT4B-265) containing an N-terminal transactivation domain was not able to reside in the nucleus, nor bind to the consensus OCT4-binding site on DNA, due to the altered configuration of the N terminal [64]. The role of OCT4B-265 in ES cells is therefore unclear, since its different intracellular localization precludes it from acting as a negative regulator of OCT4A activity. A recent study that analyzed changes to *OCT4B1* transcript levels in a gastric cancer cell line however, points to a likely role for *OCT4B1 *as an antiapoptotic factor, since cells deprived of *OCT4B1* take on a giant cell morphology or undergo apoptosis directly [60]. In ES cells, the prevalence of *OCT4B1* may also serve to manage the rapid cell cycling characteristic of this proliferative cell type. 

Interestingly, a mainly cytoplasmic pyruvate kinase, Pkm2, has previously been described as an Oct4 interactor [67] and may well be one of many Oct4B partners. Pkm2, itself one of two alternative transcripts from the* Pkm* gene, is the primary transcript at early embryonic stages [68] and was shown to bind to the POU domain of Oct4 [67]. As the large body of protein interaction data may not distinguish between Oct4 isoforms as baits, these data sets should be more carefully examined for clues that suggest functionality for OCT4B-265/Oct4B in the cytoplasm. Still, this cytoplasmic Oct4 variant might just make it into the nucleus after all—Kpna2, a protein involved in nuclear import was found to bind to the Oct4 POU domain [69]. While all Oct4 protein variants contain a nuclear localization signal, this is necessary but not sufficient for actual nuclear localization [57]. With the possibility of Oct4B transferred to a nuclear locale, albeit at lower levels than in the cytoplasm, this could serve as a self-generated negative feedback loop for the sequestration of active Oct4A, through its heterodimerization with Oct4B. 

The shorter OCT4B-190 and OCT4B-164 proteins are not typically expressed in ES cells, yet an increase in OCT4B-190 was observed on heat shock of human ES cells, and presumed to take on a protective role against apoptosis [64], thereby highlighting an additional means by which alternative transcripts function in developmental time and function, though directed from the same locus. 

Correspondingly, there is evidence that a number of Oct4 splice variants are also found in mice, although no present findings support the presence of a mouse homolog to OCT4B1 [70]. Intriguingly this may be attributed in part to the increasing evidence that suggests the nonequivalence of human and mouse ES cells. Because of the differences in growth factors and trophectoderm differentiation potential of these two cell types, it is believed that human ES cells are representative of a later epiblast stage of the developing blastocyst than mouse ES cells [71–73]. As such, it is of note that the lack of Oct4B1 in mouse ES cells might demonstrate the narrowly defined window of function for OCT4B1 in human ES cells, as supported by its possible role as a stemness marker [58]. Species-specific differences in alternative splicing may also be a contributing factor, suggesting that it is prescient to more carefully consider the model systems and likelihood of alternative splice variants in future studies.

### 4.2. Sall4

SALL4/Sall4 is a known ES cell-specific transcription factor that interacts with OCT4 and Nanog and also regulates stem cell pluripotency [74, 75]. In both mouse and human, SALL4/Sall4 exist as two splice isoforms, *Sall4a* and *Sall4b* that differ by the inclusion or exclusion of part of exon 2 and results in a different number of zinc finger domains [76, 77].* Sall4 −/−* mice fail to develop an inner cell mass (ICM), demonstrating the essentialness of one or both isoforms in early embryonic development [74, 78]. 

While most other studies have not distinguished between isoforms, *SALL4* mutations that affect both isoforms are implicated in the human Duane-Radial Ray syndrome and acute myeloid leukemia (AML) [76, 79, 80]. Interestingly, a transgenic mouse with human *SALL4B* recapitulated the features of AML, suggesting that the truncated isoform is sufficient to initiate disease. 

Chromatin immunoprecipitation experiments for Sall4a and Sall4b were informative for target genes of each of these isoforms in mouse ES cells. In line with evidence that hetero- or homodimerization of isoforms are both possible, Sall4a/b show shared as well as distinct targets in ES cells [77]. Shared targets were enriched for genes involved in developmental processes and organ morphogenesis including the essential ES cell factors Oct4, Nanog and Sox2, and Sall4a alone was targeted to a specific niche of genes involved in olfaction and sensing [77]. However, Sall4b was targeted to a larger group of genes, enriched for transcription and gene expression. In conjunction, Sall4a/b and Sall4b target genes alike were found with activating chromatin marks (H3K4me3 and H3K36me3), whereas the repressive H3K27me3 was enriched at target genes of Sall4a [77]. In the transgenic *SALL4B *mouse with AML, SALL4B expression was most evident in the initiating cancer stem cell population, but not in the chronic disease cells [76]. Sall4b is also required for proper ICM development and the maintenance of pluripotency [77, 81]. Through these animal models, it is evident that the normal contribution of this shorter isoform in development is to perpetuate self-renewal in the ICM, and in aberrant development to sustain a tumor initiating population of stem cells. Conversely, the longer Sall4a isoform appears to regulate transcription of differentiation specific genes, with bivalent chromatin marks representing active or poised genes, present only at loci that were cobound by other pluripotency factors such as Oct4 [77]. A separate Sall4a/b ChIP demonstrated that Sall4a/b binding was evident at 27% of known genes with bivalent domains—a subset of these would include Sall4a-only binding [18, 82].

Given the conservation of exons and alternative exons between human and mouse, it is likely that both Sall4 isoforms are also highly conserved across other species. Certainly the discovery of Sall4 through its homology to the *Drosophila spalt *gene [74, 83] suggests its ancestral function in organogenesis and sensory development has been retained in mammals, though this has been expanded in vertebrates through the existence of multiple Sall family genes. While exon exclusion in evolution occurs at <20% frequency in a representative human gene set, it is interesting to observe that the truncation of Exon 2 in Sall4b might have conferred additional capabilities to Sall4b for transcriptional regulation and its essential role in early development, in addition to its conserved function in cell fate differentiation with Sall4a. Despite these differences in the target genes of Sall4 brought about by an exon truncation that resulted in the loss but not ablation of all zinc finger domains, it is evident that subtle changes to domain structure through alternative splicing is able to directly impact protein function.

### 4.3. Tcf3

Tcf3 is a more recently described transcription factor that forms part of the core regulatory network present in ES cells [84–86]. As a transcriptional repressor and the effective end of the Wnt pathway, Tcf3 is able to translate extracellular stimuli into a directive for transcription through its binding to beta catenin. Genome-wide analysis of Tcf3-binding sites identified its frequent co-occupancy with some transcription factors that promulgate self-renewal, namely, Oct4 and Nanog [84–86]. Through this, both transcriptional activator(s) and repressor(s) are locked in a close counter-balancing relationship that can more sensitively regulate genes according to its cell fate requirements. While the repressive activity of human TCF3 can be attenuated through its phosphorylation [87], the gene itself also undergoes AS. Available in 2 known variants, the shorter Tcf3 (Tcf3(s)) lacks 14 a.a. present in the Groucho-binding domain of its longer twin, Tcf3(l), though both are expressed in mouse ES cells [36, 88, 89]. Through variant-specific knockdowns of Tcf3, Salomonis et al. categorized the response of 34 genes anticipated as Tcf3 target genes as defined by the available literature and looked to see if their responses differed. Sharing much similarity in domain structure, both Tcf3 variants showed overlaps in gene targets. However, despite the apparently small 14 a.a. change, it appears that Tcf3(s) and Tcf3(l) can also regulate mutually exclusive sets of genes associated with different downstream pathways. While Tcf3(s) target genes were involved with lineage differentiation, Tcf3(l) targets were more directed towards cardiac and neural development. Clearly, AS can increase the dimensionality of a protein's function, while still retaining its original purpose. Though comparisons of beta-catenin binding to each of the Tcf3 variants has not been described, it is very probable that both variants are still responsive to beta catenin, since such interactions in a domain separate from the affected Groucho binding region [90, 91]. 

Intriguingly, a recent study derived rat and human-induced pluripotent stem cells that better resembled the characteristics of mouse ES cells, including an ability to contribute to rodent chimera models when introduced into preimplantation embryos [92]. This involved the use of a GSK*β* inhibitor in the culture media, suggesting a strong role for Tcf3, as part of the Wnt pathway, in regulating pluripotency. It is plausible that the splice variants of Tcf3 observed in mouse ES cells facilitate the interconversion of ES cells between different “metastable” levels of pluripotency that ES cells are able to adopt, depending upon the available ES cell factors [93].

## 5. Conclusion

Amplifying the complexity involved in decoding a gene into a functional RNA or protein product, alternative splicing of mRNAs occurs as a post-transcriptional regulatory process that adds to the repertoire of known mechanisms for organismal complexity. Through weak associations of the mRNA with generic splicing factors, and possibly tissue-specific splicing factors, a newly transcribed mRNA may be transformed into a variety of protein and RNA products that differ in domain content, structure, and functional value, depending upon the tissue of interest. Although gene expression studies often seek to derive tissue-specific gene expression profiles as a hallmark of each tissue, these studies may not fully consider the implications of sequentially similar but alternative transcripts whose individual expression levels may not be resolved. 

In this paper, we have considered the possible functional implications of alternative splicing. As a post-transcriptional regulatory mechanism, this can result in the inclusion or exclusion of amino acid residues that can affect protein function. Because of the large number of ways in which protein function may be regulated, disruptions can occur through alternate posttranslational modifications, domain content and binding site affinities among others. RNA stability and regulation may precede or preclude the translation of such a protein, especially through the introduction of new promoter or translation start sites. Powerfully, AS is observed through evolution and first understood as a means of increasing signal nodes and complexity despite the relative similarity of DNA content between species of recent divergence. While approximately 3/4 of all known genes undergo AS, only a third of these are estimated to show cross-species conservation. A study that considered the extent of AS for a single gene between primate species suggests that the frequency of such events is not conserved and instead may correlate with life expectancy. While such correlations may be more case appropriate than prescriptive for the diverse spectrum of known genes, it is interesting to consider how transcript variety can have seemingly profound implications on a species' life cycle and the extent of an individual's environmental interactions. Additional studies demonstrate that tissue-specific genes are subject to AS more often than ubiquitously expressed “housekeeping genes”. In view of the common developmental pathways shared between mammals, embryonic stem cell biology has been instrumental in providing insight into early developmental stages. Here, we highlight 3 key ES cell transcription factors in human and mouse that are essential for self-renewal and also show that these factors undergo AS. While transcription factors such as Oct4 are generally perceived to be expressed specifically in the ICM, and to a lesser extent in primordial germ cells or their *in vitro *equivalents, it is increasingly evident that our previous notions of what constitutes “expression” needs to be more clearly distinguished, and that low levels of alternative transcripts may be present in alternative cell types, but remain unknown. These transcripts can serve to discriminate one species from the next. Although the minority of AS products are conserved between species, such products are often associated with biologically important, core pathways [43].In this paper, a number of these AS variants appear to show such behavior, in association with their essential role in ES cell renewal. At this point, much research in ES cell biology has not delved significantly to consider the impact of alternative transcripts in modulating or expanding the function of key ES cell factors. We anticipate that future studies that examine the possibility of splice variants in more detail might bring forth new evidence to distinguish ES cell mechanisms and species differences more clearly.

## Abbreviations

ESC/ES Cells: Embryonic Stem Cells
ICM: Inner Cell Mass
iPS Cells: Induced Pluripotent Stem Cells
UTR: Untranslated Region
AS: Alternative Splicing
ChIP: Chromatin Immunoprecipitation
miRNA: Micro-RNAs
NMD: Nonsense mediated decay.
